# Characteristics of patients presenting to the vascular emergency department of a tertiary care hospital: a 2-year study

**DOI:** 10.1186/1756-0500-4-481

**Published:** 2011-11-03

**Authors:** Ioannis Kotsikoris, Theofanis T Papas, Nikolaos Papanas, Dimitrios Maras, Paraskevi Tsiantula, Polyvios Pavlidis, Maria Andrikopoulou, Stamatia Kotsiou, Efstratios Maltezos, Nikolaos Bessias

**Affiliations:** 1 Department of Vascular Surgery, Red Cross Hospital, Athens, Greece; 2 Second Department of Internal Medicine, Democritus University of Thrace, Alexandroupolis, Greece

**Keywords:** Emergency, Health care resources, Primary health care, vascular disease

## Abstract

**Background:**

The structure of health care in Greece is receiving increased attention to improve its cost-effectiveness. We sought to examine the epidemiological characteristics of patients presenting to the vascular emergency department of a Greek tertiary care hospital during a 2-year period. We studied all patients presenting to the emergency department of vascular surgery at Red Cross Hospital, Athens, Greece between 1^st ^January 2009 and 31st December 2010.

**Results:**

Overall, 2452 (49.4%) out of 4961 patients suffered from pathologies that should have been treated in primary health care. Only 2509 (50.6%) needed vascular surgical intervention.

**Conclusions:**

The emergency department of vascular surgery in a Greek tertiary care hospital has to treat a remarkably high percentage of patients suitable for the primary health care level. These results suggest that an improvement in the structure of health care is needed in Greece.

## Background

The emergency department of vascular surgery in a tertiary care hospital is a pivotal constituent of the Greek health care system [1,2]. It is designed for the treatment of patients with various vascular pathologies, such as vascular trauma, ruptured aortic aneurysm or acute arterial occlusion, that prompt urgent hospital admission and vascular surgery. At the same time, it is frequented by patients suffering from chronic vascular diseases that could be treated at the primary health care level or in the outpatient vascular clinics [1-3]. Such conditions include, for instance, chronic intermittent claudication, varicose veins, lymphoedema, or lower extremity infections. The aim of this study was to examine the epidemiological characteristics of patients presenting to the vascular emergency department of a Greek tertiary care hospital during a 2-year period.

## Results

We studied the epidemiological characteristics of all patients presenting to the vascular emergency department of Red Cross Hospital, Athens, Greece between 1^st ^January 2009 and 31^st ^December 2010. Patient records were studied retrospectively. Information retrieved included permanent address, presenting symptom, mode of attendance (private vehicle, ambulance, physician referral, referral from other hospitals of Athens or the rest of the country), underlying disease and treatment. In total, 4961 patients (3780 men, 1181 women, mean age 63 years) presented to the vascular emergency department for diagnosis and treatment. Of these, 4018 (81%) were residents of Athens and 943 (19%) were residents of the rest of the country. Overall, 2833 (57.1%) came by private vehicle following their own decision without any physician referral, 546 (11.2%) came by ambulance from the region of Athens. The remaining 1582 (31.9%) patients came with a referral letter either by a general practitioner or by a physician of another specialty from our own hospitals emergency department or by a by physician from another hospital, in which there was no vascular surgery department.

Pathologies that should have been treated at the primary health care level or in the outpatient vascular clinics were noted in 2452 patients (49.4%). Such pathologies included chronic vein insufficiency, painful varicose veins, ankle oedema due to congestive heart failure, lymphatic oedema, lower extremity infection, intermittent claudication and chronic diabetic foot ulcers (Table 1). Of these patients, 1962 (80%) had typical clinical manifestations that would have enabled the general practitioner to diagnose and manage the condition, while in 490 (20%) clinical manifestations were less clear. These latter patients could have been diagnosed and treated, however, via ordinary patient referral to in vascular outpatient clinics.

**Table 1:** Pathologies that might have been treated at the primary health care level or in vascular outpatient clinics.

Pathology	Patients
Chronic vein insufficiency	206
Painful varicose veins	170
Ankle oedema due to congestive heart failure	303
Lymphoedema	189
Lower extremity infection	514
Intermittent claudication	667
Chronic diabetic foot ulcers	403
**TOTAL**	**2452**

Patients who actually needed vascular surgical interventions were 2509 (50.6%). Emergency revascularisation was performed in 556 patients (11.2%) (Table 2). Finally, 85% of presenting patients were less affluent (immigrants, less well-off subjects, or those with chronic drug abuse).

**Table 2:** Pathologies prompting emergency revascularisation in our patients.

Pathology	Patients
Ruptured abdominal aortic aneurysm	42
Upper limb embolism	33
Lower extremity embolism	61
Graft thrombosis	31
Acute lower extremity ischaemia prompting revascularisation with graft placement	44
Acute abdominal aortic thrombosis	6
Pseudoaneurysm	12
Vascular trauma	26
Lower extremity amputation due to gangrene	21
Vascular access complications in end-stage renal disease	251
Miscellaneous	29
**TOTAL**	**556**

## Discussion

The major finding of this study is that almost half of patients presenting to the vascular emergency department could have been treated at the primary health care level or via ordinary patient referral to vascular outpatient clinics. This agrees with reports from USA, Canada and Europe [3-6] and may be explained by the structure of the Greek health care system.

The Greek health care system is a mixed public and private health care system, which is fully accessible to the entire population, whereas at the same time an overdeveloped private sector is also available and plays an important role in the overall health care. In Greece, health care services are provided by a) the National Health System (public hospitals and health centres in rural and semi-urban areas), which is free; b) the health insurance funds (health centres with physicians), which are also free; c) the private sector (hospitals, diagnostic centres, private practitioners) that require payment. Health insurance funds are public schemes financed by employees, employers and the public budget. It is obligatory for the entire workforce (including their families) to be insured in one of the 8 different health insurance funds, selection depending on professional status [2]. Importantly, every patient is entitled to visit any hospital he or she prefers without any physician referral as a prerequisite. This unique liberty reflects the absence of well-organised and reliable primary health care. Especially the less affluent parts of society (immigrants, poorer subjects and those with chronic drug abuse) make use of this liberty.

This also applies to vascular emergency departments [1,2]. Indeed, first aid in public hospitals in Greece and many other countries is free for all, irrespective of nationality, professional status, duration of residence in the country and identification as to whether residence is legal or not [2,4,7] Secondly, anybody can visit emergency departments at any time, without prior appointment, which does not necessitate absence from work [1,2,8] Thirdly, emergency departments are accessible without bureaucracy, thereby minimising potential linguistic, cultural and other barriers [1,2,4,7,8].

Of note, the majority of presenting patients belonged to the less affluent parts of society (immigrants, less well-off subjects, or those with chronic drug abuse). Such subjects resort to emergency departments because they cannot afford private health care and they may even lack insurance, so that access to insurance-covered resources (outpatient clinics in hospitals, health centres) is very difficult[1,2]. This agrees with previous reports from other countries as well [9,10].

Our findings suggest that the structure of the Greek health care system needs to be improved. Three changes appear to be particularly important. First, the role of primary health care needs to be strengthened. This involves increasing the number of general practitioners but also providing them with more time, resources and authority to diagnose and provide first treatment. Secondly, some control over patient referral to specialised centres is desirable. Such control may be expected to enable timely patient allocation to the most suitable care level, preventing financial and personnel exhaustion in tertiary care centres. Thirdly, we need to revise legislation relating to health care facilities offered to immigrants and less affluent subjects, in order to enable them to use primary health care and outpatient clinics for simple health problems rather than frequenting emergency departments. These, and possibly other changes, may require time and effort from health policy makers to be implemented, but they have potential to contribute to a more rational use of emergency departments and other health care facilities.

## Conclusions

Our findings suggest that half of patients presenting to the vascular outpatient department should have been treated at the primary health care level or in the outpatient vascular clinics. Indeed, only 1 out of 10 patients actually needed urgent revascularisation. Of note, the majority of presenting patients belonged to the less affluent parts of society. Consequently, there is increased cost and personnel use for the vascular outpatient department, as well as for the national ambulance service. Thus, improved organisation of the national health care system is required.

## Competing interests

The authors declare that they have no competing interests.

## Authors' contributions

IK, conception and design, acquisition of data, analysis and interpretation of data, helped to draft the manuscript; TTP, conception and design, acquisition of data, analysis and interpretation of data, Corresponding author; NP, coordination and helped to draft the manuscript; DM, conception and design, acquisition of data, analysis and interpretation of data; PT, analysis and interpretation of data, acquisition of data; PP, acquisition of data; MA, acquisition of data; SK, design and coordination and helped to draft the manuscript; EM, coordination and provision of useful insights to the manuscript; NB, conception, design, coordination, analysis and interpretation of data, and helped to draft the manuscript. All authors read and approved the final manuscript.
